# Effects of Exercise Training on Anabolic and Catabolic Hormones with Advanced Age: A Systematic Review

**DOI:** 10.1007/s40279-021-01612-9

**Published:** 2021-12-22

**Authors:** Hassane Zouhal, Ayyappan Jayavel, Kamalanathan Parasuraman, Lawrence D. Hayes, Claire Tourny, Fatma Rhibi, Ismail Laher, Abderraouf Ben Abderrahman, Anthony C. Hackney

**Affiliations:** 1 M2S, Laboratoire Mouvement, Sport, Santé, EA 1274, Université Rennes, 35000 Rennes, France; 2 Institut International des Sciences du Sport (2I2S), 35850 Irodouer, France; 3 SRM College of Physiotherapy, SRM Institute of Science and Technology, SRM Nagar, Kattankulathur, Kanchipuram, Chennai, TN 603203 India; 4 Institute of Clinical Exercise and Health Science, School of Health and Life Sciences, University of the West of Scotland, Lanarkshire Campus, Glasgow, G72 0LH UK; 5 EA 3832 CETAPS, University of Rouen, Rouen, France; 6 Faculty of Medicine, Department of Anesthesiology, Pharmacology and Therapeutics, The University of British Columbia, Vancouver, Canada; 7 ISSEP Ksar-Said, University of La Manouba, Tunis, Tunisia; 8 Department of Exercise and Sport Science, Department of Nutrition, University of North Carolina, Chapel Hill, NC USA

## Abstract

**Background:**

Ageing is accompanied by decreases in physical capacity and physiological regulatory mechanisms including altered hormonal regulation compared with age-matched sedentary people. The potential benefits of exercise in restoring such altered hormone production and secretion compared to age-matched physically inactive individuals who are ageing remains unclear.

**Objectives:**

The aim of this systematic review was to summarise the findings of exercise training in modulating levels of ostensibly anabolic and catabolic hormones in adults aged > 40 years.

**Methods:**

We searched the following electronic databases (to July 2021) without a period limit: Cochrane Library, PubMed, Science Direct, Scopus, SPORTDiscus and Web of Science. Additionally, a manual search for published studies in Google Scholar was conducted for analysis of the ‘grey literature’ (information produced outside of traditional commercial or academic publishing and distribution channels). The initial search used the terms ‘ageing’ OR ‘advanced age’ OR ‘old people’ OR ‘older’ OR elderly’ AND ‘anabolic hormones’ OR ‘catabolic hormones’ OR ‘steroid hormones’ OR ‘sex hormones’ OR ‘testosterone’ OR ‘cortisol’ OR ‘insulin’ OR ‘insulin-like growth factor-1’ OR ‘IGF-1’ OR ‘sex hormone-binding globulin’ OR ‘SHBG’ OR ‘growth hormone’ OR ‘hGH’ OR ‘dehydroepiandrosterone’ OR ‘DHEA’ OR ‘dehydroepiandrosterone sulfate (DHEA-S)’ AND ‘exercise training’ OR ‘endurance training’ OR ‘resistance training’ OR ‘ strength training’ OR ‘weight-lifting’ OR ‘high-intensity interval training’ OR ‘high-intensity interval exercise’ OR ‘high-intensity intermittent training’ OR ‘high-intensity intermittent exercise’ OR ‘interval aerobic training’ OR ‘interval aerobic exercise’ OR ‘intermittent aerobic training’ OR ‘intermittent aerobic exercise’ OR ‘high-intensity training’ OR ‘high-intensity exercise’ OR ‘sprint interval training’ OR ‘sprint interval exercise’ OR ‘combined exercise training’ OR ‘anaerobic training’. Only eligible full texts in English or French were considered for analysis.

**Results:**

Our search identified 484 records, which led to 33 studies for inclusion in the analysis. Different exercise training programs were used with nine studies using endurance training programs, ten studies examining the effects of high-intensity interval training, and 14 studies investigating the effects of resistance training. Most training programs lasted ≥ 2 weeks. Studies, regardless of the design, duration or intensity of exercise training, reported increases in testosterone, sex hormone-binding globulin (SHBG), insulin-like growth factor-1 (IGF-1), human growth hormone (hGH) or dehydroepiandrosterone (DHEA) (effect size: 0.19 < *d* < 3.37, small to very large) in both older males and females. However, there was no consensus on the effects of exercise on changes in cortisol and insulin in older adults.

**Conclusion:**

In conclusion, findings from this systematic review suggest that exercise training increases basal levels of testosterone, IGF-1, SHBG, hGH and DHEA in both male and females over 40 years of age. The increases in blood levels of these hormones were independent of the mode, duration and intensity of the training programs. However, the effects of long-term exercise training on cortisol and insulin levels in elderly people are less clear.

## Key Points

**Table:** 

For basal concentrations of ostensibly anabolic hormones, exercise training produced trivial to very large increases in testosterone, small to moderate increases in insulin-like growth factor-1 (IGF-1), small to very large increases in human growth hormone (hGH), trivial to very large increases in insulin, and small to large increases in dehydroepiandrosterone sulphate (DHEA-S) in adults aged > 40 years.
Small to large increases were observed for basal sex hormone-binding globulin (SHBG).
For the ostensibly catabolic hormone cortisol, effects ranged from a small decrease to a very large increase. Moderate effects and large effects were observed for the other corticosteroids cortisone and corticosterone, respectively.
Observed alterations to the hormonal mileu were not readily related to participant age or acute training programme variables, or chronic training variables (e.g. intensity, volume, frequency).

## Introduction

Ageing is characterized by decreases in physical capacity that are related to a loss of muscle mass and decreased muscle contraction velocity [1] and maximum strength, which accelerate in particular after 50 years of age [2, 3]. According to Korhonen et al. [4], strength and muscle volume peaks around ~ 30 years of age and decreases by 15% per decade from age 50 onwards, until 70 years of age, when strength is ~ 30% of that of a 30-year-old [5, 6]. This decrease in muscle strength is due in large part to decreases in the contractile properties of muscle [5, 7], especially in the number of fast-twitch type II muscle fibers [5, 8, 9].

Loss of physical function and hormonal status are important determinants of health and longevity in older adults [10–13]. Concentrations of anabolic hormones are altered with advancing age, as shown by yearly 1% decreases in testosterone in about 50% of men ≥ 30 years of age (from peak values at 20 years of age) [14], suggesting changes in anabolic hormones start from the fourth decade of life [15, 16]. The fall in blood testosterone levels during ageing diminishes its anabolic effects in skeletal muscle, and so negatively impacts neuromuscular performance [17], muscle mass and bone mineral density [18].

Ageing alters metabolism and degradation of hormones, especially in those individuals with decreased liver or kidney function [19]. In addition, ageing reduces target cell hormone receptor number, affinity and signal transduction [20, 21]. Moreover, anabolic hormones such as testosterone inhibit the secretion of cortisol, diminishing glucocorticoid-mediated catabolism of skeletal muscle, meaning testosterone has both anabolic and anti-catabolic effects [22–24]. Nearly 98% of circulating testosterone is bound to sex hormone-binding globulin (SHBG) and albumin [25], both of which are also altered by the ageing process [26]. As the concentration of SHBG increases with age, the level of free (unbound) and bioavailable (bound loosely to albumin) testosterone decreases, so that there is less testosterone available for tissue uptake [27]. Interestingly, there are similar patterns of androgenic hormone decline (*andropause*) and somatrotropic hormone decline (*somatopause*); the latter also have anabolic actions [14]. Specifically, human growth hormone (hGH) and its main downstream protein, insulin-like growth factor-1 (IGF-1), decrease with advanced age [14]. Secretion of hGH decreases by ~ 14% per decade after 20 years of age [14], and reaches, by the age of ~ 60 years, half of the hGH secretion of younger counterparts (20–30 years) [28]. The main stimulated protein downstream of hGH, IGF-1, as already noted, also decreases with age (~ 10% per decade) [28]. IGF-1 is also anabolic in nature, increases cell proliferation, cell differentiation and energy metabolism, and prevents apoptosis [29].

Although endocrine dysregulation is associated with advanced age, it is difficult to attribute this alteration to age exclusively, as physical activity and exercise also influence the hormonal mileu [19]. Age is associated with physical inactivity [30], which can influence the age-endocrine dysregulation relationship. While ageing per se may not cause endocrinological dysregulation, age-associated increases in sedentary behaviour could [31–34]. Exercise is a non-pharmacological strategy to counteract come of the physiological changes that occur with age, including endocrine changes [19, 35–40].

Exercise exerts well-known health-promoting cardioprotective effects [41], with recent meta-analytical evidence demonstrating running activities were associated with a 30% reduction in cardiovascular mortality [42]. This emphasizes the importance of physical activity for health, supporting the recent narrative by the UK government that identified a curvilinear dose–response relationship between physical activity and health outcomes [43]. Moreover, several reports or opinion pieces suggest exercise may be a countermeasure to human biological ageing [44]. Thus, exercise and physical activity ameliorate many deleterious effects of chronological ageing on multiple physiological systems. There are some reports that lifelong exercisers are more phenotypically younger in terms of endocrinological profile than their sedentary counterparts, and exercise interventions result in a ‘younger’ hormonal profile than before undertaking exercise [45]. Therefore, it appears consistent physical exercise may be required to maintain endocrine function with ageing. However, before exercise can be proposed as a viable countermeasure to endocrinological dysregulation, it is important to consider the existing literature in terms of methodologies, quality of research and heterogeneity, and conduct a systematic review of available literature. To the best of our knowledge, only one narrative review [19] and two book chapters [46, 47] have reviewed the effects of physical exercise on changes in anabolic and catabolic hormones in older adults. Therefore, it seemed prudent to conduct a systematic review of the effects of various exercise training protocols on ostensibly anabolic and catabolic hormones in people aged > 40 years with normal body mass.

## Methods

### Eligibility Criteria

Population, Intervention, Comparison, Outcome and Study design (PICOS) criteria were used for inclusion of studies in this review (see Table 1) [48]. This systematic review included original studies (randomized or non-randomized) for which the full texts were available and that performed interventions with exercise training, included 2 or more weeks of follow-up, and involved subjects who were aged between 40 and 85 years. We included studies that involved one or both sexes, and specifically evaluated blood levels of any of the following hormones: total testosterone, cortisol, insulin, IGF-1, SHBG, hGH, dehydroepiandrosterone (DHEA) and dehydroepiandrosterone sulfate (DHEA-S) before and after exercise.

**Table 1:** PICOS (participants, interventions, comparisons, outcomes, study design)

PICOS component	Details
Participants	Healthy humans aged: > 40 and < 85 years
Interventions	Exercise training with two or more weeks of follow-up
Comparisons	Control group/Untrained participants
Outcomes	Physical performances, anabolic/catabolic hormone responses
Study designs	nRCTs, nRnCTs and RCTs

*nRCT* non-randomized controlled trial, *nRnCT* non-randomized non-controlled trial, *RCT* randomized controlled trial

Duplicate publications or sub-topics of included studies [e.g., studies involving co-morbidities or pathologies, and studies linking exercise to nutritional interventions (e.g., nutrition counselling, balanced or hypocaloric diets, and supplements)] or pharmacological agents were all excluded to reduce confounding factors [49]. Studies involving individuals with overweight or obese BMIs (BMI ≥ 25 kg/m^2^] were also excluded [49].

### Literature Search Strategy

This systematic review is reported in accordance with the Preferred Reporting Items for Systematic Reviews and Meta-Analyses (PRISMA) statement and the Cochrane Handbook for Systematic Reviews of Interventions [50]. The study protocol was registered (CRD42019138269) in the International Prospective Register of Systematic Reviews (PROSPERO) platform.

We searched the following electronic databases (to July 2021) without a period limit: Cochrane Library, PubMed, Science Direct, Scopus, SPORTDiscus and Web of Science. Additionally, a manual search for published studies in Google Scholar was conducted for analysis of the ‘grey literature’ (information produced outside of traditional commercial or academic publishing and distribution channels). The initial search used the terms ‘ageing’ OR ‘advanced age’ OR ‘old people’ OR ‘older’ OR elderly’ AND ‘anabolic hormones’ OR ‘catabolic hormones’ OR ‘steroid hormones’ OR ‘sex hormones’ OR ‘testosterone’ OR ‘cortisol’ OR ‘insulin’ OR ‘insulin-like growth factor-1’ OR ‘IGF-1’ OR ‘sex hormone-binding globulin’ OR ‘SHBG’ OR ‘growth hormone’ OR ‘hGH’ OR ‘dehydroepiandrosterone’ OR ‘DHEA’ OR ‘dehydroepiandrosterone sulfate (DHEA-S)’ AND ‘exercise training’ OR ‘endurance training’ OR ‘resistance training’ OR ‘ strength training’ OR ‘weight-lifting’ OR ‘high-intensity interval training’ OR ‘high-intensity interval exercise’ OR ‘high-intensity intermittent training’ OR ‘high-intensity intermittent exercise’ OR ‘interval aerobic training’ OR ‘interval aerobic exercise’ OR ‘intermittent aerobic training’ OR ‘intermittent aerobic exercise’ OR ‘high-intensity training’ OR ‘high-intensity exercise’ OR ‘sprint interval training’ OR ‘sprint interval exercise’ OR ‘combined exercise training’ OR ‘anaerobic training’. Only eligible full texts in English or French were considered for analysis.

### Study Selection and Data Extraction

Three authors independently performed searches in the electronic databases, and disagreements were resolved by consensus. The literature search strategies used for all databases are available in the supporting information.

**Fig. 1:**
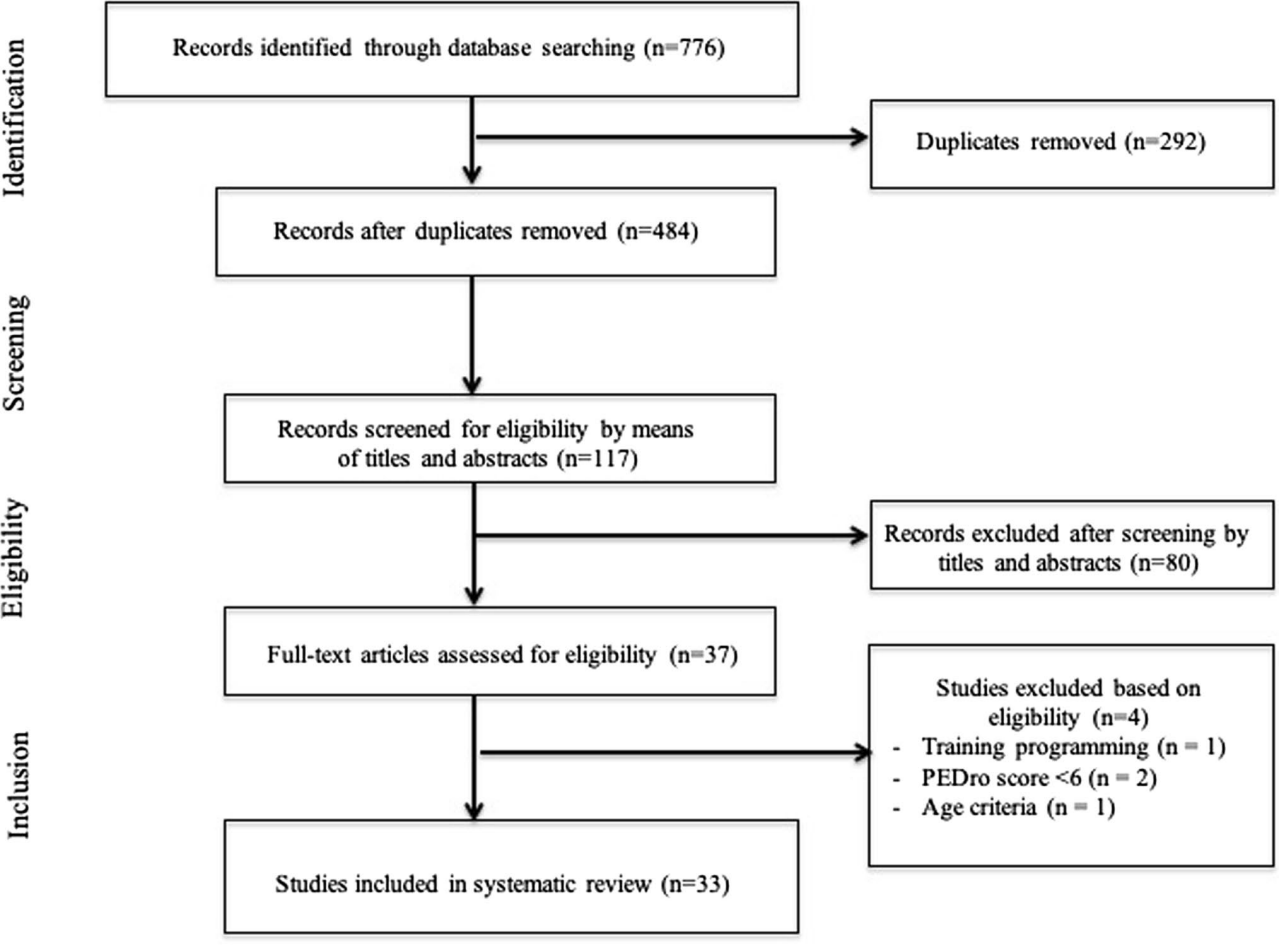
Selection process for research articles (*n* = 33) included in this systematic review. Adapted version of the recommendations in the PRISMA (Preferred Reporting Items for Systematic Reviews and Meta-Analyses) statement [43]

The data-collection process is shown in Fig. 1 [51]. Titles and abstracts of selected articles were independently assessed by two researchers (HZ and AJ). The reviewers were not blinded to the authors, institutions or journals associated with the studies. Abstracts that provided insufficient information on inclusion and exclusion criteria were retrieved for full-text analysis. Furthermore, the researchers independently analysed the full text and determined the eligibility of the studies, and disagreements were resolved by consensus. The agreement rate between the reviewers was 97% for the eligibility criteria of the study.

Corresponding authors of publications were contacted to avoid duplicate-counting of participants or to clarify questions about the methods where necessary. The corresponding authors were also contacted to provide data that may not have been included in the publications. Two researchers (HZ and AJ) independently performed the data extraction, and disagreements were resolved by consensus. Data were extracted for pre- and post-training hormone levels.

### Assessment of Risk of Bias

The quality of studies was assessed using the Physiotherapy Evidence Database (PEDro) scale (http://www.pedro.fhs.usyd.edu.au), which has good reliability and validity [52]. The PEDro scale has 11 possible points that examine external validity (criterion 1) and internal validity (criteria 2–9) of controlled trials, and also enable determination of whether there was sufficient statistical information for interpreting results (criteria 10–11). The items of the scale are: (1) eligibility criteria were specified; (2) subjects were randomly allocated to groups; (3) allocation was concealed; (4) groups were similar at baseline; (5) subjects were blinded; (6) therapists who administered the treatment were blinded; (7) assessors were blinded; (8) measures of key outcomes were obtained from more than 85% of subjects; (9) data were analysed by intention to treat; (10) statistical comparisons between groups were conducted; and (11) point measures and measures of variability were provided. The first criterion is not included in the final score. Moreover, because of the nature of the physical activity interventions, where patient and therapy blinding and allocation are unlikely, the total score a trial could receive was limited to eight points. A cut-off of six points on the PEDro scale signified high-quality studies, as this has been reported to be sufficient to determine high-quality versus low-quality studies [52]. The studies were evaluated by two experienced investigators (HZ and AJ), and in the event of disagreement a third reviewer (ACH) was invited to further review the findings.

### Data Analysis

The percent change (Δ%) was calculated (if not available in the study) for each study to evaluate the magnitude of the effects using the following equation:

$$\Delta \% = \left( {{\text{Mpost}} - {\text{Mpre}}} \right)/{\text{Mpre}} \times 100$$

where Mpost represents the mean value after (acute exercise or long-term of training) and Mpre represents the baseline mean value.

Effect sizes (ES) were computed to present standardized effects of acute and long-term training on the outcome variables (e.g., hormones and physical performance). The ES was calculated with Cohen’s *d* [53] by dividing the raw ES (difference in means) by the pooled standard deviations:

$${\text{ES}} = \left( {{\text{Cohen}}^{\prime}{\text{s}}\;d} \right) = \left. {\left( {M1 - M2} \right)/{\text{SD}}\;{\text{pooled}}} \right]$$

Values for ES were defined as trivial (< 0.2), small (0.2–0.6), moderate (0.6–1.2), large (1.2–2.0) and very large > 2 [54]. Results for each outcome variable are presented with number of observations (*N*), Δ% and ES. Data analysis was processed using SigmaStat 3.5 software (Systat, Inc, San Jose, CA, USA). The ES and Δ% were analysed in studies where sufficient data were available. A significant difference was indicated when the 95% confidence interval (CI) of the ES did not overlap zero.

## Results

### Study Selection

Our search identified 484 studies related to the effects of exercise on hormone levels in adults over 40 years of age (Fig. 1)*.* After screening of titles, abstracts and full texts, 33 studies were selected for inclusion in our final analysis, and the characteristics of these long-term studies are summarised in Table 2. The 33 studies were carried out in different countries from five continents (Africa, North America, Europe, Asia and Australia). Fifteen studies investigated only male subjects, ten studied female subjects exclusively, while eight studies investigated both males and females.

**Table 2:** Characteristics of studies that examined the effect of acute and chronic exercises on anabolic and catabolic hormones

Study	Year	PEDro scale	Population/sex/sample size	Sample size	Country	Age, years (mean ± SD) or age range	Characteristics of exercise training	Duration (weeks)
Friedenreih et al. [69]	2019	8	Post-menopausal women	396	Canada	59.4 ± 4.9	Moderate and high-intensity training	52
Vaczi et al. [59]	2014	7	Older men	16	Hungary	65.7 ± 5.3	Stretch shortening cycle and eccentric training	10
Im et al. [10]	2019	7	Older women	25	Korea	69.4 ± 2.9	Yoga and Korean dance	12
Søgaard et al. [77]	2018	6	Older men and women	22	Denmark	63 ± 1	High-intensity interval training	6
Ahtiainen et al. [60]	2011	7	Older men	35	Finland	61 ± 5	Heavy resistance exercise	21
Ahtiainen et al. [61]	2015	7	Older men	13	Finland	70 ± 2	Heavy resistance exercise	52
Banitalebi et al. [70]	2018	9	Older women	48	Iran	67.4 ± 1.4	Resistance and endurance training	12
Consitt et al. [76]	2016	7	Young and older	20	USA	19–29 and 57–82	Endurance and strength training	12
DiPietro et al. [63]	2008	7	Older women	20	USA	77 ± 6	Aerobic training and strength training	36
Glintborg et al. [62]	2013	10	Older males	54	Denmark	68	Strength training	12
Ha et al. [72]	2018	7	Older women	20	North Korea	73 ± 2.8	Combined resistance training and aerobic training	12
Hayes et al. [63]	2015	7	Older men	48	Scotland	61 ± 5	Low- to medium- and high-intensity training	6
Hayes et al. [90]	2015	6	Sedentary aged men	22	UK	62 ± 2	High-intensity training	6
Kim et al. [56]	2017	7	Older men and women	555	USA	51	Moderate physical activity	52
Krishnan et al. [65]	2013	7	Premenopausal women	28	USA	46.7 ± 3.3	Aerobic and resistance training	24
Micielska et al. [73]	2019	6	Healthy inactive women	33	Poland	45 ± 13	High-intensity circuit training	5
Motiani et al. [55]	2017	7	Sedentary men and women	26	Finland	45–55	Moderate-intensity interval training	2
Nunes et al. [66]	2019	7	Post-menopausal women	34	Brazil	64.2	Resistance training	16
Ogawa et al. [74]	2010	6	Older women	21	Japan	85.0 ± 4.5	Resistance training	12
De Guia et al. [94]	2019	6	Older men	43	Denmark	46.5 ± 3.0	Aerobic and resistance training	12
Praksch et al. [78]	2019	7	Older women	60	Hungary	67.4 ± 5	Home-based walking and aerobic training	12
Ramos et al. [75]	2016	7	Elderly men and women	66	Australia	58 ± 7	MICT and high-intensity training	16
Sato et al. [67]	2014	6	Older men	19	Japan	67.2 ± 1.8	Resistance training	12
Sellami et al. [15]	2016	7	Moderately trained late adult men	36	Tunisia	40.7 ± 1.8	Combined sprint and resistance training	13
Sellami et al. [16]	2018	7	Moderately trained late adult men	40	Tunisia	40 ± 2	Combined sprint and resistance training	13
Walker et al. [68]	2015	7	Older men	18	USA	63.7 ± 3	Resistance training	20
Yamada et al. [79]	2015	7	Community-dwelling older men and women	222	Japan	76.3 ± 5.9	Walking exercise and nutrition	24
Bermon et al. [80]	1999	6	Sedentary and trained older adults	32	France	70.1 ± 1	Strength training	8
Bennefoy et al. [82]	1999	6	Community-dwelling older adults	32	France	69.7 ± 2.2	Physical activity	2
Craig et al. [58]	1989	6	Older men	9	USA	62.8 ± 0.7	Progressive resisted exercise	12

*PEDro scale* physiotherapy evidence database scale, *F* female, *M* male, *M/F* male and female, *x* times, *W* weeks, *H* hour, *min* minutes, *Others* other methods of intervention beyond the physical activity, *BMI* body mass index, *SD* standard deviation

A total of 2211 participants (age range = 40–85 years) underwent exercise-training programs and completed the studies. The 33 studies used different exercise-training protocols, with nine studies using endurance-training programs, ten studies examining the effects of high-intensity interval training (HIIT) and 14 studies investigating the effects of resistance training. The training duration lasted at least 2 weeks [4], but several studies used ~ 12 weeks, and four studies used a 52-week exercise protocol [55, 56]. Studies were generally classified as ‘high-quality’ studies (mean 6.9 in the PEDro scale score) (Table 3).

**Table 3:** Physiotherapy evidence database (PEDro) score of the included longitudinal studies

Study	Year	Eligibility criteria	Randomized allocation	Blinded allocation	Group Homogeneity	Blinded subjects	Blinded therapists	Blinded assessor	Drop out ≥ 15%	Intention-to-treat analysis	Between-group comparison	Point estimates and variability	PEDro sum
Aerobic—endurance training													
Bennefoy et al. [82]	1999	●	○	○	●	○	○	●	●	○	●	●	6
Consitt et al. [76]	2016	●	●	○	●	○	○	●	●	○	●	●	7
DiPietro et al. [71]	2008	●	●	○	●	○	○	●	●	○	●	●	7
Kim et al. [56]	2017	●	●	○	●	○	○	●	●	○	●	●	7
Krishnan et al. [65]	2014	●	●	○	●	○	○	●	●	○	●	●	7
Praksch et al. [78]	2019	●	●	○	●	○	○	●	●	○	●	●	7
Yamada et al. [79]	2015	●	●	○	●	○	○	●	●	○	●	●	7
Im et al. [10]	2019	●	●	○	●	○	○	●	●	○	●	●	7
High-intensity interval training													
Friedenreich et al. [69]	2019	●	●	○	●	○	○	●	●	●	●	●	8
Vaczi et al. [59]	2014	●	●	○	●	○	○	●	●	○	●	●	7
Søgaard et al. [77]	2019	●	○	○	●	○	○	●	●	○	●	●	6
Hayes et al. [63]	2015	●	●	○	●	○	○	●	●	○	●	●	7
Hayes et al. [90]	2017	●	○	○	●	○	○	●	●	○	●	●	6
Micielska et al. [73]	2019	●	○	○	●	○	○	●	●	○	●	●	6
Motiani et al. [55]	2017	●	●	○	●	○	○	●	●	○	●	●	7
Ramos et al. [75]	2016	●	●	○	●	○	○	●	●	○	●	●	7
Sellami et al. [15]	2016	●	●	○	●	○	○	●	●	○	●	●	7
Resistance training													
Ahtiainen et al. [60]	2011	●	●	○	●	○	○	●	●	○	●	●	7
Ahtiainen et al. [61]	2015	●	●	○	●	○	○	●	●	○	●	●	7
Banitalebi et al. [70]	2018	●	●	●	●	●	○	●	●	○	●	●	9
Bermon et al. [80]	1999	●	○	○	●	○	○	●	●	○	●	●	6
Craig et al. [58]	1989	●	○	○	●	○	○	●	●	○	●	●	6
Glintborg et al. [62]	2013	●	●	●	●	●	●	●	●	○	●	●	10
Ha et al. [72]	2018	●	●	○	●	○	○	●	●	○	●	●	7
Nunes et al. [66]	2019	●	●	○	●	○	○	●	●	○	●	●	7
Ogawa et al. [74]	2010	●	○	○	●	○	○	●	●	○	●	●	6
Sato et al. [67]	2014	●	○	○	●	○	○	●	●	○	●	●	6
Sellami et al. [16]	2018	●	●	○	●	○	○	●	●	○	●	●	7
Walker et al. [68]	2015	●	●	○	●	○	○	●	●	○	●	●	7

### Total Testosterone

Twelve studies investigated the effects of training on testosterone concentrations in older adults [16, 57–67] (Table 4). Irrespective of the exercise protocol (type of exercise, duration or intensity of exercise training), these studies all reported increases in basal total testosterone in both male and female participants (effect size: 0.19 < *d* < 3.37, small to very large).

**Table 4:** Effects of training on total testosterone concentrations in elderly people

Reference(s)	Year	Intervention	Population		Outcomes	Effect size
			Age	Sex		
Ahtiainen et al. [60]	2011	Heavy resistance exercise	61 ± 5	Male	Testosterone ↑	0.38
Ahtiainen et al. [61]	2015	Heavy resistance exercise	70 ± 2	Male	Testosterone ↑	1.99
Craig et al. [58]	1989	Progressive resistance training	62.8 ± 0.7	Male	Testosterone ↑	0.40
Glintborg et al. [62]	2013	Strength training	68–78	Male	Testosterone ↑	0.90
Hayes et al. [63]	2015	Low to medium and high intensity training	61 ± 5	Male	Testosterone ↑	0.22
Hayes et al. [90]	2015	High-intensity interval training	62 ± 2	Male	Testosterone ↑	0.24
Krishnan et al. [65]	2014	Aerobic and resistance training	46.7 ± 3.3	Female	Testosterone ↑	0.19
Nunes et al. [66]	2019	Resistance training	64.2	Female	Testosterone ↑	0.29
Sato et al. [67]	2014	Resistance training	67.2 ± 1.8	Male	Testosterone ↑	3.37
Sellami et al. [16]	2018	Combined sprint and resistance training	40 ± 2	Male	Testosterone ↑	1.60
Vaczi et al. [59]	2014	Stretch shortening cycle and eccentric training	65.7 ± 5.3	Male	Testosterone ↑	0.32
Walker et al. [68]	2015	Resistance training	63.7 ± 3	Male	Testosterone ↑	0.39

↑ Indicates increase, ↓ indicates decrease

### Cortisol

Nine studies investigated the effects of exercise training on cortisol in older adults (Table 5). Six studies reported increases in basal cortisol concentrations [15, 16, 58, 64, 67, 68] (effect size: 0.27 < *d* < 2.69, small to very large), while three studies observed decreases [62, 63, 69] (effect size: 0.27 < *d* < 0.46, small).

**Table 5:** Effects of training on cortisol concentrations in elderly people

Reference(s)	Year	Intervention	Population		Outcomes	Effect size
			Age	Sex		
Banitalebi et al. [70]	2018	Resistance and endurance training	67.3 ± 1.4	Female	Cortisol ↓	0.27
Friedenreich et al. [69]	2019	Moderate- and high-intensity training	59.4 ± 4.9	Female	Cortisol ↑ Cortisone ↑ Corticosterone ↑	2.69 0.61 1.12
Hayes et al. [63]	2015	Low- to medium- and high-intensity training	61 ± 5	Male	Cortisol ↓	0.39
Hayes et al. [90]	2015	High-intensity training	62 ± 2	Male	Cortisol ↓	0.46
Nunes et al. [66]	2019	Resistance training	64.2 ± 1.2	Female	Cortisol ↑	0.31
Sellami et al. [15]	2016	High-intensity sprint training and strength training	40.7 ± 1.8	Male	Cortisol ↑	0.98
Sellami et al. [16]	2018	Combined sprint and resistance training	40 ± 2	Male	Cortisol ↑	0.27
Vaczi et al. [59]	2014	Stretch shortening cycle and eccentric training	65.7 ± 5.3	Male	Cortisol ↑	0.37
Walker et al. [68]	2015	Resistance training	63.7 ± 3	Male	Cortisol ↑	0.38

↑ Indicates increase, ↓ indicates decrease

### Insulin

The effects of long-term training on insulin concentrations in older adults are summarised in Table 6. Nine studies [15, 64, 65, 70–74] reported decreased basal insulin (effect size: 0.04 < *d* < 2.30, small to very large) and four studies [4, 69, 75, 76] observed increased concentrations (effect size: 0.32 < *d* < 0.56, small).

**Table 6:** Effects of training on insulin concentrations in elderly people

Reference(s)	Year	Intervention	Population		Outcomes	Effect size
			Age	Sex		
Banitalebi et al. [70]	2018	Resistance and endurance training	67.3 ± 1.4	Female	Insulin ↑	0.56
Consitt et al. [76]	2016	Endurance and strength training	67 ± 3.3	Male and female	Insulin ↑	0.32
DiPietro et al. [71]	2008	Aerobic training and strength training	77 ± 6	Female	Insulin ↓	0.08
Guia et al. [94]	2019	High intensity interval training	62.3 ± 4.1	Male	Insulin ↓	1.90
Ha et al. [72]	2018	Combined resistance training and aerobic training	73.0 ± 2.8	Female	Insulin ↓	0.22
Krishnan et al. [65]	2014	Aerobic and resistance training	46.7 ± 3.3	Female	Insulin ↓	0.79
Micielska et al. [73]	2019	High-intensity circuit training	45 ± 13	Female	Insulin ↓	0.34
Motiani et al. [55]	2017	Moderate-intensity continuous training and high-intensity training	50.0 ± 3.6	Male	Insulin ↑	0.22
Nunes et al. [66]	2019	Resistance training	64.2 ± 1	Female	Insulin ↓	0.29
Ogawa et al. [74]	2010	Resistance training	85.0 ± 4.5	Female	Insulin ↓	2.30
Ramos et al. [75]	2016	Moderate-intensity continuous training and high-intensity training	58 ± 7	Male and female	Insulin ↓	0.04
Sellami et al. [15]	2016	High-intensity sprint training and strength training	40.7 ± 1.8	Male	Insulin ↓	0.60
Søgaard et al. [77]	2019	High-intensity interval training	63 ± 1	Male and female	Insulin ↑	0.32

↑ Indicates increase, ↓ indicates decrease

### Insulin-Like Growth Factor-1 (IGF-1)

Nine studies investigated the effects of training on IGF-1 concentrations in older adults (Table 7). Among these, eight studies reported significant increases in IGF-1 in both elderly males and females (effect size: 0.27 < *d* < 1.03, small to moderate) [65, 66, 69, 72, 77–80] and one study reported decreased IGF-1 [73] (effect size: 1.06).

**Table 7:** Effects of training on insulin-like growth factor-1 (IGF-1) concentrations in elderly people

Reference(s)	Year	Intervention	Population		Outcomes	Effect size
			Age	Sex		
Banitalebi et al. [70]	2018	Resistance and endurance training	67.3 ± 1.4	Female	IGF-1 ↑	0.27
Bennefoy et al. [82]	1999	Physical activity	69.7 ± 2.2	Male and female	IGF-1 ↑	0.46
Bermon et al. [80]	1999	Resistance training	70.1 ± 1.0	Male and female	IGF-1 ↑	0.97
Micielska et al. [73]	2019	High-intensity circuit training	45 ± 13	Female	IGF-1 ↑	0.32
Nunes et al. [66]	2019	Resistance training	64.2	Female	IGF-1 ↑	0.26
Ogawa et al. [74]	2010	Resistance training	85.0 ± 4.5	Female	IGF-1 ↓	1.06
Praksch et al. [78]	2019	Home-based walking and aerobic training	67.4 ± 5	Female	IGF-1 ↑	0.28
Sato et al. [67]	2014	Resistance training	67.2 ± 1.8	Male	IGF-1 ↑	1.03
Yamada et al. [79]	2015	Walking exercise and nutrition	76.3 ± 5.9	Male and female	IGF-1 ↑	0.51

↑ Indicates increase, ↓ indicates decrease

### Sex Hormone-Binding Globulin (SHBG)

Studies of the effects of training on SHBG concentrations in older adults are summarised in Table 8. Six studies [15, 55, 60–63] reported increases in basal SHBG concentrations in elderly women and men irrespective of the type, duration and intensity of exercise training (effect size: 0.25 < *d* < 1.68, small to large).

**Table 8:** Effects of training on sex hormone-binding globulin (SHBG) concentrations in elderly people

Reference(s)	Year	Intervention	Population		Outcomes	Effect size
			Age	Sex		
Ahtiainen et al. [61]	2015	Heavy resistance exercise	70 ± 2	Male	SHBG ↑	0.25
Glintborg et al. [62]	2013	Strength training	68	Male	SHBG ↑	0.32
Hayes et al. [63]	2015	Low- to medium- and high-intensity training	61 ± 5	Male	SHBG ↑	0.38
Hayes et al. [90]	2017	High-intensity training	62 ± 2	Male	SHBG ↑	0.43
Kim et al. [56]	2017	Moderate physical activity	51	Male and female	SHBG ↑	0.32
Sellami et al. [16]	2018	Combined sprint and resistance training	40 ± 2	Male	SHBG ↑	1.68

↑ Indicates increase, ↓ indicates decrease

### Growth Hormone (hGH)

Only four studies investigated the effects of long-term training on basal hGH concentrations in older adults [67, 69, 73, 81] (Table 9). These studies reported increases in hGH in response to physical training (effect size: 0.29 < *d* < 2.58, small to very large).

**Table 9:** Effects of training on human growth hormone (hGH) concentrations in elderly people

Reference(s)	Intervention	Population		Outcomes	Effect size
		Age	Sex		
Banitaleb et al. [70]	Resistance and endurance training	67.3 ± 1.4	Female	GH ↑	2.58
Craig et al. [58]	Progressive resistance training	62.8 ± 0.7	Male	GH ↑	0.34
Im et al. [10]	Yoga and Korean dance	69.3 ± 2.9	Female	GH ↑	0.74
Walker et al. [68]	Resistance training	63.7 ± 3	Male	GH ↑	0.29

↑ Indicates increase, ↓ indicates decrease

### Dehydroepiandrosterone (DHEA] and Dehydroepiandrosterone Sulfate (DHEA-S)

Studies examining the effects of long-term training on basal DHEA concentrations in older adults are summarised in Table 10. Six studies [10, 55, 64, 66, 78, 82] reported increases in DHEA in older males and females (effect size: 0.37 < *d* < 1.71, small to large). Only one study [65] reported a decrease (effect size: 0.28, small) in DHEA in post-menopausal women in response to 16 weeks of resistance training.

**Table 10:** Effects of training on dehydroepiandrosterone (DHEA) and dehydroepiandrosterone sulfate (DHEA-S) concentrations in elderly people

Reference(s)	Intervention	Population		Outcomes	Effect size
		Age	Sex		
Boxer et al. [83]	DHEA supplements, aerobics and yoga	76.4 ± 6.2	Female	DHEA-S ↑	1.37
Im et al. [10]	Yoga and Korean dance	69.3 ± 2.9	Female	DHEA-S ↑	0.98
Kim et al. [56]	Moderate physical activity	51	Male and female	DHEA-S ↑	0.37
Krishnan et al. [65]	Aerobic and resistance training	46.7 ± 3.3	Female	DHEA-S ↑	0.41
Nunes et al. [66]	Resistance training	64	Female	DHEA-S ↓	0.28
Sato et al. [67]	Resistance training	67.2 ± 1.8	Male	DHEA-S ↑	1.71
Yamada et al. [79]	Walking exercise and nutrition	76.3 ± 5.9	Male and female	DHEA-S ↑	0.55

↑ Indicates increase, ↓ indicates decrease

## Discussion

**Fig. 2:**
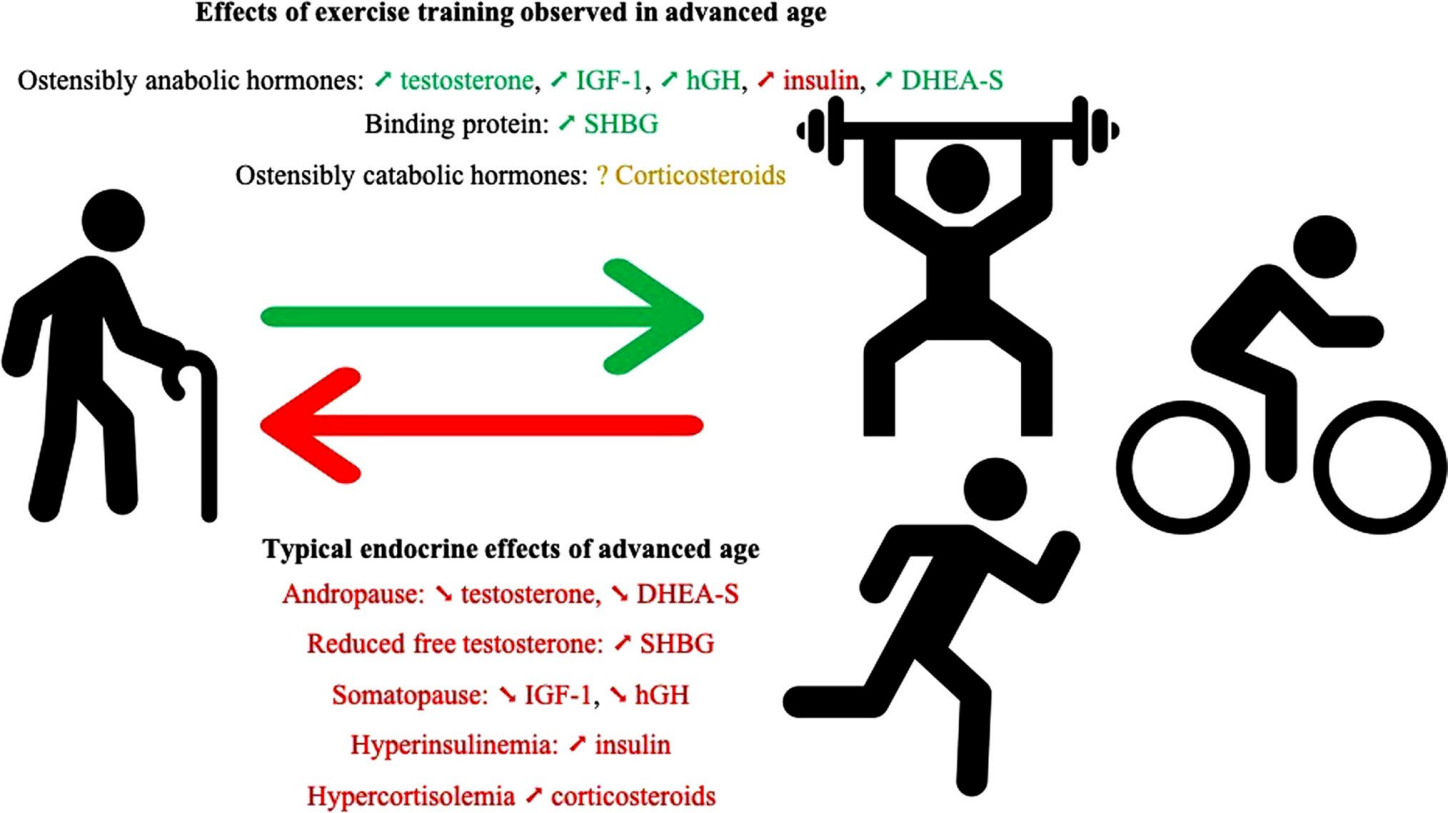
Top: Summary of the main effects of exercise on hormones discussed in this review. Bottom: Summary of the main effects of ageing on hormones discussed in this review. *IGF-1* insulin-like growth factor-1, *hGH* human growth hormone, *DHEA-S* dehydroepiandrosterone sulfate, *SHBG* sex hormone-binding globulin. ➚ indicates increase; ➘ indicates decrease

This systematic review indicates that exercise training increases basal total testosterone, IGF-1, SHBG, hGH, DHEA and DHEA-S in males and females ≥ 40 years of age. Effects of exercise on blood hormones occurred regardless of the type, duration and intensity of training programs, with the exception of a lack of consensus on the effects of long-term exercise training on cortisol and insulin responses in older adults (Fig. 2).

### Effect of Exercise on Testosterone Levels

Exercise tended to produce small to large increases in total testosterone, supporting the supposition that exercise is the most convenient non-pharmacological means of increasing testosterone production and concomitantly preventing muscle loss in the elderly [60]. Different forms of exercise training can increase testosterone [60], although this finding is not ubiquitous [32, 83]. For example, 6 weeks of progressive resistance exercise increased muscle testosterone levels in the elderly, due to increased muscle steroidogenesis [66]. This corresponded to increased blood free testosterone, although total testosterone was not reported as commonly measured by other investigations, which may explain the divergency of results. Herbert et al. [33] previously reported that exercise increased free testosterone but not total testosterone levels [33]. Preconditioning exercise (10% increase) and HIIT (7% increase) combined to increase total testosterone by 17% in previously sedentary males, but only increased free testosterone by 5% [63]. Thus, the fraction of testosterone measured may result in different findings between studies, as supported by a recent meta-analysis of exercise-induced testosterone changes that concluded that free testosterone, rather than total testosterone, was more likely to change following resistance exercise [83].

The findings of this systematic review suggest exercise increases blood testosterone in both males and females, although several studies suggest men were more likely to benefit from exercise training in terms of increased total testosterone [59–61, 63]. Yet, benefits of exercise training in both sexes stretch beyond steroidogenesis, and include effects such as muscle mass regeneration, weight loss, disability prevention and prevention of sarcopenia [64, 65]. Differences in exercise training, such as type, intensity, frequency and duration, can potentially affect testosterone levels and muscle mass, with exercise volume being a critical component [84]. Results from this review demonstrate different forms of exercise (such as resistance training, HIIT, aerobic training) aid in maintenance of blood testosterone levels and muscle mass of the elderly. Levels of free testosterone, representing the fraction available for tissue uptake, are increased by exercise when total testosterone levels increase with no changes in SHBG levels [16]. There is much interest in regulation of testosterone levels in the aged as low testosterone is associated with many non-communicable diseases such as diabetes [85], cardiovascular disease [86], Alzheimer’s disease [87], dementia [88], obesity [89] and ultimately mortality [86]. Evidence of increases in circulating testosterone (particularly the free fraction) by non-pharmacological means (e.g., exercise) has important implications for patients and clinicians [66, 67, 90]. Nevertheless, it remains unclear if increases in blood testosterone through exercise: (a) exceed inherent analytical and biological variability [91], and (b) exert benefits on ageing physiology in addition to the other effects of exercise.

### Effects of Exercise on Cortisol

The results of our analysis indicate that effect sizes have qualitative differences [i.e., the direction of the effect (increase or decrease)] in the various studies, ranging from a small decrease in cortisol to a very large increase, indicating an inconsistency in findings. Cortisol, corticosterone, cortisone and 11-deoxycortisol are key biomarkers of stress, particularly of acute stress [68]. Exercise training reduces stress in the elderly and often decreases basal cortisol and raises testosterone levels [58]. Recent studies report that post-training basal blood cortisol levels are decreased relative to non-exercise populations, and that exercise-induced changes in cortisol are unrelated to the volume, type and duration of exercise [68, 69]. A study by Hayes et al. [63] reported no improvements in blood cortisol levels after 12 weeks of training (of which 6 weeks was HIIT), although other reports indicated that increases in blood cortisol levels are not related to the type of exercise. It is possible that a single exercise bout may not be sufficient to cause persistent changes in adrenal function; however, benefits are likely if the exercise intensity is sufficient (~ 60% maximal oxygen uptake), which is common in younger adults [15]. Another possibility is that age-related changes in the hypothalamus-pituitary axis may alter responses to exercise [14].

### Effect of Exercise on DHEA and DHEA-S

Small to very large increases of DHEA and DHEA-S post-training were generally observed in our analysis, although the number of studies was relatively small. Men’s DHEA-S responses were also greater than those of women [55]. DHEA levels decreased around 65 years of age and were associated with reduced muscle strength in males but not in females [81]. However, a significant decline in the circulating androgen DHEA-S between the ages 20 and 50 years was associated with the normal ageing process for women [64]. We observed that exercise tends to maintain blood levels of DHEA in both men and women. Even a single bout of exercise has demonstrated immediate increases in circulating androgen levels (testosterone and DHEA-S) [64]. DHEA hormonal levels are in general positively correlated with normal physical activity, involvement in sport and aerobic ability [93]. The effects of exercise training on DHEA-S levels differ in males and females [55]. Several studies have shown that serum DHEA levels in older adults are increased significantly by exercise training [66, 78]. Recent evidence suggests that increased levels of DHEA-S during the menopausal transition can double cardiovascular disease risk and diabetes mellitus [10].

### Effect of Exercise on Insulin

We found that exercise training reduces blood insulin levels in older adults in most studies, with four studies showing small to moderate increases [55, 70, 76, 77]. Effect sizes ranged from small to very large, with some variability in the findings. These divergent effects did not seem to be related to study duration, as those studies with the largest positive effects [73, 91] were only 12 weeks in duration. This corroborates a recent study [94] in which no changes were noted to insulin levels measured after 12 weeks of training in lifelong exercisers or lifelong sedentary older adults. Small to moderate effects were observed in the previously sedentary group only, suggesting that participant selection may have contributed to the variability we found. Insulin sensitivity decreases with age and can lead to insulin resistance and type 2 diabetes mellitus [70, 75]. Skeletal muscle dysfunction and disuse associated with ageing is a primary cause of impaired glucose absorption and reduced mitochondrial oxidation [75]. Skeletal muscles are responsible for > 60% of glucose metabolism [72], and thus exercise in older adults is an important regulator of glucose metabaolism. HIIT improves insulin sensitivity, body composition and cardiovascular health [76], and several studies suggest the increase in insulin sensitivity due to exercise training is directly proportional to exercise intensity and volume [70, 71], although there is also evidence that combined training may be more efficacious than one form of training alone [64, 69–71].

### Effect of Exercise on IGF-1

Levels of IGF-1 were ubiquitously increased in the all the studies we analysed, with small to large effect sizes being evident. IGF-1 plays an important role in muscle development and insulin sensitivity [65], and changes in IGF-1 are correlated with cardiovascular disease risk and mortality [77]. IGF-1 levels decrease during ageing (i.e., the somatopause), but several exercise types can affect the synthesis of IGF-1 [65, 66, 72, 77]. Increased IGF-1 levels cause downstream improvements in insulin activity [65, 66, 77]. Resistance training reportedly causes greater increases in IGF-1 relative to endurance training [65, 69]. Combined training with different exercise sequences did not affect IGF-1 [69]. When comparing concurrent training with interval training, concurrent training produced greater increases in IGF-1 levels [69]. Yet, high-intensity circuit training also produced significant elevations of IGF-1 that were accompanied by improvements in the homeostatic model assessment index of insulin resistance (HOMA1-IR) [72]. One important issue to consider from these data is the interpretation of increased IGF-1 levels. For example, Herbert et al. [33] reported increased IGF-1 following HIIT in previously sedentary older masters athletes, who had higher levels of IGF-1 than sedentary individuals, causing these authors to propose increased IGF-1 in older men was a positive physiological adaptation. Conversely, Hayes et al. [95] concluded IGF-1 decreases were a positive physiological adaptation following resistance training. These authors hypothesized that IGF-1 entered muscle tissue to exert downstream hypertrophic effects during periods of muscle building (i.e., anabolism). Muscle hypertrophy is not the only response to perturbations in IGF-1, with other effects of IGF-1 possible in participants who exercise.

### Effect of Exercise on SHBG

Our analysis indicates that levels of SHBG in older adults were universally increased by exercise. This glycoprotein binds to androgens and oestrogens and its levels increase with age whilst testosterone decreases, resulting in lower levels of bioavailable and free testosterone available for biological effects following tissue uptake. Therefore, it is imperative to measure both SHBG and testosterone levels to obtain a better understanding of androgenic status. In this context, Hayes et al. [63] previously reported increases in total testosterone and SHBG levels, with no alterations in free testosterone levels [62]. There were larger increases in total testosterone SHBG when HIIT was used as the exercise protocol, suggesting increases in free testosterone [63]. Therefore, it is important to distinguish which hormone fraction is being measured in such studies. Low SHBG suggests a high androgenic nature in women, which is inversely related with adiposity and SHBG levels [55].

### Effect of Exercise on Human Growth Hormone

We found only four studies that measured hGH, all which reported increases in hGH having effect sizes from small to very large [10, 58, 68, 70]. One possible reason for the limited number of studies measuring hGH may be its short biological half-life, making it more pragmatic to measure IGF-1, the main downstream protein of hGH secretion [68]. When comparing other forms of exercise, resistance training causes the largest increases in hGH levels in older adults [67]. Ageing accelerates the reduction of hGH secretion in both sexes, with females experiencing steeper declines than males [67, 69, 77]. Combined exercise programs such as resistance and endurance increase hGH levels [69].

## Conclusion

In conclusion, exercise increases the levels of anabolic hormones in older adults, although the clinical significance of these alterations remains uncertain. It is apparent that exercise exerts anti-ageing effects on several physiological systems, but whether these effects are mediated by the endocrine system is unclear at this time. Nonetheless, we recommend that exercise should be considered as a first-line treatment for endocrine dysfunction as it improves several changes of the hormonal regulation that occur with ageing. Future investigations may wish to address the effects of exercise on hormonal concentrations in middle-aged individuals as this is typically where age-associated hormonal milieu alterations may begin to manifest. In the present review, regrettably we found that too few original articles were conducted in participants aged 40–60 years, so additional articles in this age category would have allowed us to examine age differences and would have permitted practical applications for each age category.

## Data Availability

All data supporting the findings of this study are available in this published article.
